# Ethanol Inhibits Aflatoxin B_1_ Biosynthesis in *Aspergillus flavus* by Up-Regulating Oxidative Stress-Related Genes

**DOI:** 10.3389/fmicb.2019.02946

**Published:** 2020-01-17

**Authors:** Yaoyao Ren, Jing Jin, Mumin Zheng, Qingli Yang, Fuguo Xing

**Affiliations:** ^1^College of Food Science and Engineering, Qingdao Agricultural University, Qingdao, China; ^2^Key Laboratory of Agro-products Quality and Safety Control in Storage and Transport Process, Ministry of Agriculture and Rural Affairs/Institute of Food Science and Technology, Chinese Academy of Agricultural Sciences, Beijing, China

**Keywords:** aflatoxin B_1_, *Aspergillus flavus*, transcriptome, RNA-seq, oxidative stress, ethanol

## Abstract

As the most carcinogenic, toxic, and economically costly mycotoxins, aflatoxin B_1_ (AFB_1_) is primarily biosynthesized by *Aspergillus flavus* and *Aspergillus parasiticus*. Aflatoxin biosynthesis is related to oxidative stress and functions as a second line of defense from excessive reactive oxygen species. Here, we find that ethanol can inhibit fungal growth and AFB_1_ production by *A. flavus* in a dose-dependent manner. Then, the ethanol’s molecular mechanism of action on AFB_1_ biosynthesis was revealed using a comparative transcriptomic analysis. RNA-Seq data indicated that all the genes except for *aflC* in the aflatoxin gene cluster were down-regulated by 3.5% ethanol. The drastic repression of aflatoxin structural genes including the complete inhibition of *aflK* and *aflLa* may be correlated with the down-regulation of the transcription regulator genes *aflR* and *aflS* in the cluster. This may be due to the repression of several global regulator genes and the subsequent overexpression of some oxidative stress-related genes. The suppression of several key aflatoxin genes including *aflR*, *aflD*, *aflM*, and *aflP* may also be associated with the decreased expression of the global regulator gene *veA*. In particular, ethanol exposure caused the decreased expression of stress response transcription factor *srrA* and the overexpression of bZIP transcription factor *ap-1*, C_2_H_2_ transcription factors *msnA* and *mtfA*, together with the enhanced levels of anti-oxidant enzymatic genes including *Cat*, *Cat1*, *Cat2*, *CatA*, and Cu, Zn superoxide dismutase gene *sod1*. Taken together, these RNA-Seq data strongly suggest that ethanol inhibits AFB_1_ biosynthesis by *A. flavus* via enhancing fungal oxidative stress response. In conclusion, this study served to reveal the anti-aflatoxigenic mechanisms of ethanol in *A. flavus* and to provide solid evidence for its use in controlling AFB_1_ contamination.

## Introduction

*Aspergillus flavus* is a saprophytic fungus being often found in mildewed grains, grain products, and other moldy organic matter, and causes the wastage of several important agricultural crops (Wild and Gong, 2010; Liang et al., 2015). In addition, this fungus is an opportunistic human and animal pathogen causing aspergillosis diseases (Amaike and Keller, 2010). It is more important to notice that this fungus can produce aflatoxins (AFs), the most potent natural carcinogen and toxic compounds ever characterized (Da Rocha et al., 2014). In 1993, AFs are classified as a Class 1 carcinogen by the (International Agency for Research on Cancer [IARC], 1993, 2002), and were estimated to induce up to 28% of the total global cases of hepatocellular carcinoma (HCC) (Wu, 2014; Liu et al., 2017). AFs are mainly produced by *A. flavus* and *Aspergillus parasiticus*, and the former is the predominant aflatoxigenic species of contaminated foods and feeds in China (Xing et al., 2017). The most common AF-contaminated food and feed are aflatoxin B_1_ (AFB_1_), B_2_, G_1_, and G_2_ (Bennett and Klich, 2003). Among AFs, AFB_1_ is the most potent natural carcinogen and toxic compound known (Squire, 1981; Marin et al., 2013). Therefore, it is urgent to develop simple, economical, and effective ways to control *A. flavus* and subsequent AF contamination in food and feed, especially during storage and processing.

As we all know, ethanol is an inhibitor of the growth of bacteria and fungi (Ma et al., 2019). Previous studies showed that the accumulation of ethanol inhibited yeast cell growth and viability, affected the integrity of the cell membrane, and inactivated cellular enzymes, resulting in cell death during fermentation (Gibson et al., 2007; Kim et al., 2016; Ma et al., 2019). Ma et al. (2019) indicated that ethanol stress induced an obvious suppression of *Aspergillus oryzae* growth and conidia formation, and the inhibitory effect increased with ethanol concentration. As a general cell toxic substance, ethanol disturbed many cellular processes, such as irregular nuclei, the aggregation of scattered vacuoles, the increase of unsaturated fatty acid, and the overexpression of related fatty acid desaturases (Ma et al., 2019).

Transcriptional sequencing (RNA-Seq) has been widely applied to study lots of eukaryotic transcriptomes because of high sensitivity, low false-positive rates, and broad expression range coverage (Wilhelm et al., 2008; Wang et al., 2009; Lin et al., 2013; Lv et al., 2018). For *A. flavus*, this technology has been used to explore the mechanism of action of water activity (*a*_*w*_) and temperature on fungal growth and AF production (Yu et al., 2011; Zhang et al., 2014; Bai et al., 2015). Moreover, it also has been used to decipher the inhibitory mechanism of 5-azacytidine (5-AC) (Lin et al., 2013), 2-phenylethanol (Chang et al., 2015), eugenol (Lv et al., 2018), gallic acid (Zhao et al., 2018), and cinnamaldehyde (Wang et al., 2019) on *A. flavus* growth and AF formation. The objective of this study was to determine transcriptomic changes in *A. flavus* treated with ethanol and untreated samples using RNA-Seq technology. In particular, ethanol’s molecular mechanism of action on AF biosynthesis was elucidated. This study may pave a way for further understanding the inhibitory mechanism of action of ethanol on AF formation at the transcriptomic level.

## Materials and Methods

### Chemicals, Fungal Strain, and Growth Conditions

Ethanol (100% purity) was purchased from Beijing Chemical Works (Beijing, China). Chromatographic grade methanol and acetonitrile were purchased from Thermo Fisher Scientific (Waltham, MA, United States). The AFB_1_ standard was purchased from Sigma-Aldrich (Sigma-Aldrich Chemicals, St. Louis, MO, United States).

The *A. flavus* strain NRRL3357 was obtained from Dr. Wenbing Yin, Institute of Microbiology, Chinese Academy of Sciences, and was maintained in the dark on potato dextrose agar (PDA, purchased from Hopebio, Qingdao, China) at 4°C. Conidia suspension of 1 × 10^7^ conidia/ml was prepared by surface washing PDA culture with 0.1% Tween-80 solution.

In order to investigate the effect of ethanol on *A. flavus* growth, after filtering with 0.22 μm filters, ethanol was added into the autoclaved PDA medium to obtain the final concentrations of 2, 2.5, 3, 3.5, 4, 5, and 6%. As the control group, PDA plates without ethanol were prepared. Then, 5 μl of 10^3^–10^7^ conidia/ml suspension was inoculated on PDA medium and incubated at 28°C for 7 days. A requisite amount of the ethanol was added to the autoclaved yeast extract sucrose (YES, purchased from Hopebio, Qingdao, China) broth to obtain the final concentrations of 1, 2, 2.5, 3, 3.5, and 4%. Then, 100 μl of 10^7^ conidia/ml suspension was added to 100 ml of YES broth containing different concentrations of ethanol. The control cultures were treated similarly but without ethanol. After incubation at 28°C and 180 rpm/min in the dark for 7 days, fungal mycelia were collected. Each treatment was conducted in triplicate.

### Determination of Mycelia Weights and AF Production

The dry weights of fugal mycelia were determined according to the method described by Yamazaki et al. (2007). AFB_1_ levels were determined according to the method described by Liang et al. (2015). It was extracted with acetonitrile:water (84:16) mixture from 10 ml of culture broth and purified using a ToxinFast immunoaffinity column (Huaan Magnech Biotech, Beijing, China). AFB_1_ was quantified using an HPLC system with a fluorescence detector (Agilent 1220 Infinity II System, Santa Clara, CA, United States) and a post-column derivation system (Huaan Magnech Biotech), and a TC-C18 column (250 mm × 4.6 mm, 5 μm particle size; Agilent, Santa Clara, CA, United States). The mean recovery of AFB_1_ (1–100 ng/ml) was 95.3% ± 7.5%, and the lowest detection limit was 1 ng/ml.

### Preparation of cDNA Libraries, RNA Sequencing, and Data Analysis

RNA extraction, cDNA libraries preparation, and data analysis were conducted according to the methods described by Lv et al. (2018). An Illumina^®^ HiSeq 4000^TM^ system (San Diego, CA, United States) was used to sequence the cDNA libraries. The RNA-seq data have been deposited in the NCBI Sequence Read Archive with accession code SRP217458.

The EST sequencing, rRNA sequencing, and assembling were performed using the programs TopHat v2.0.12 (Trapnell et al., 2009), Bowtie2 (Langmead et al., 2009), and Cufflinks, respectively. The transcription levels of genes were normalized using the FPKM values (Trapnell et al., 2010). The differential expression of genes was analyzed using DEseq software (Anders and Huber, 2010). The significant differentially expressed genes were identified as log_2_Ratio ≥ 1 and *q* < 0.05 between these compared samples (Zhao et al., 2018).

### Quantitative Reverse Transcription QRT-PCR Analysis of AF Biosynthesis Genes

All genes in the AF biosynthesis cluster were analyzed using QRT-PCR according to the methods described by Lv et al. (2018).

## Results

### Inhibitory Effect of Ethanol on Fungal Growth and AFB_1_ Production by *A. flavus*

**FIGURE 1:**
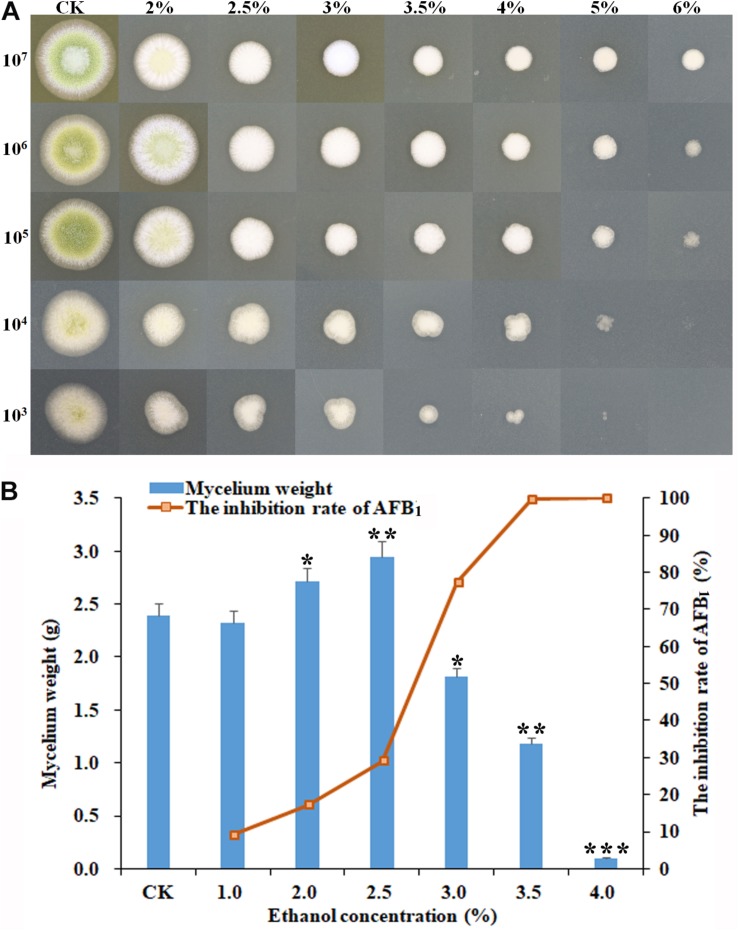
Effect of ethanol on the mycelial growth and AFB_1_ production of *A. flavus* NRRL3357. **(A)** The inhibitory effect of ethanol at different concentrations (from 0 to 6%) on mycelial colonies on PDA plates by inoculating the serial dilutions of *A. flavus* conidia (from 10^7^ to 10^3^) at 48 h post-treatment. **(B)** The mycelia biomass of *A. flavus* and the inhibition rate of AFB_1_ in YES broth at 120 h post-treatment. ^∗^*p* < 0.05; ^∗∗^*p* < 0.01; ^∗∗∗^*p* < 0.001.

As shown in Figure 1, some significant morphological changes of mycelial colonies were observed in *A. flavus* treated with ethanol compared with the control. The diameters of *A. flavus* colonies appeared much smaller than the control after treatment with 2–6% ethanol in a dose-dependent manner, and the mycelia growth was completely inhibited by 6% ethanol when the initial concentration was ≤10^4^ conidia/ml (Figure 1A). In YES broth, as shown in Figure 1B, the dry mycelia weights of *A. flavus* appeared much lower in 3.5–4.0% ethanol application compared to the control. AFB_1_ production was significantly inhibited by 3.0–4.0% ethanol with the inhibition rate up to 99.8%. Interestingly, the mycelia weight was higher in 2.0–2.5% ethanol application compared to the control, but the AFB_1_ level was obviously decreased. Taken together, these findings suggested that ethanol significantly inhibited fungal growth and AFB_1_ production by *A. flavus*. Moreover, the suppressive effect increased with the rising levels of ethanol.

### Overall Transcriptional Response Profile of *A. flavus* to Ethanol

To decipher the potential inhibitory mechanism of ethanol on *A. flavus* growth and AFB_1_ biosynthesis, a transcriptome analysis was carried out. Via RNA-seq, averagely 47.81 million, 46.01 million, and 49.49 million raw reads were generated from control, 2.5 and 3.5% of ethanol treatment samples, respectively. After filtering, 46.30 million, 44.85 million, and 47.34 million clean reads were obtained, and 96.09, 93.99, and 94.32% of total clean reads from control, 2.5 and 3.5% ethanol group were aligned to reference sequences. Based on the FPKM values with FDR ≤ 0.05 and Log2Ratio ≥ 1 or ≤ −1, 2240 and 2434 differentially expression genes (DEGs) were down-regulated and up-regulated under 2.5% ethanol treatment compared with control. Under 3.5% ethanol treatment, 2636 and 3105 DEGs were down-regulated and up-regulated compared with control, respectively. Compared with 2.5% ethanol, 973 and 1547 DEGs were down-regulated and up-regulated under 3.5% ethanol treatment, respectively.

### Functional and Pathway Analysis of DEGs

**FIGURE 2:**
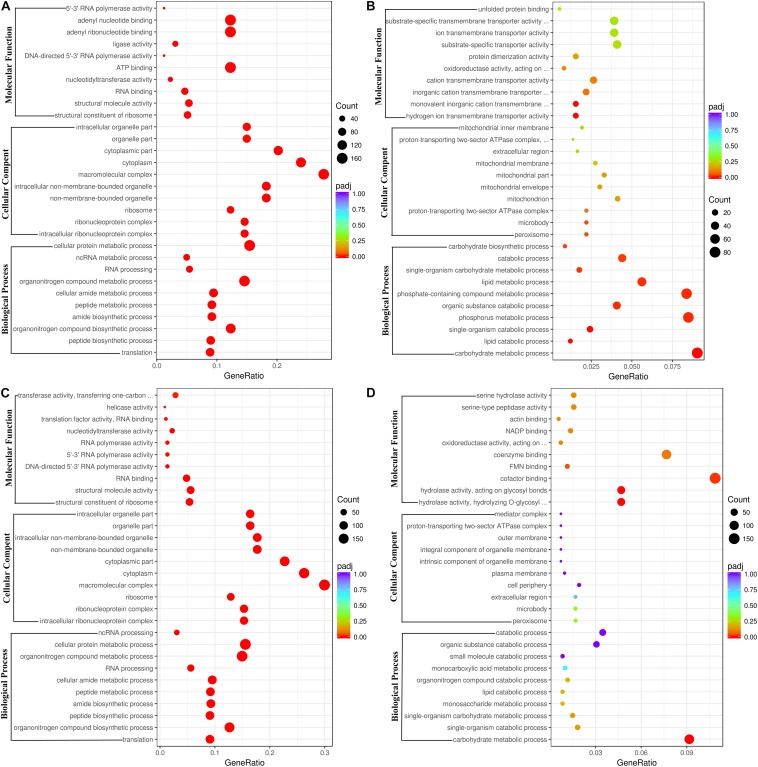
Go functional classification of down-regulated **(A,C)** and up-regulated **(B,D)** DEGs. **(A,B)** The ordinate means with 2.5% ethanol treatment. **(C,D)** The ordinate means with 3.5% ethanol treatment. The size of the plot represents the number of DEGs in one GO term; the color of the plot close to red represents more significant enrichment.

The DEGs between the ethanol treatment and the control provided an important clue to decipher the molecular mechanism of action of ethanol on fungal growth and AFB_1_ production. The functions, metabolic pathways and interactions of these DEGs were analyzed using GO and KEGG enrichment analysis. Figure 2A showed the top 30 enriched functional categories of 2240 down-regulated DEGs in *A. flavus* treated with 2.5% ethanol. Therein, cellular protein metabolic process, organonitrogen compound metabolic process, organonitrogen compound biosynthetic process, etc. were obvious enrichment terms in the biological process. Adenyl nucleotide binding, adenyl ribonucleotide binding, ATP binding, etc. were the main terms in molecular function. For the up-regulated DEGs in the 2.5% ethanol group (Figure 2B), carbohydrate metabolic process, phosphorus metabolic process, phosphate-containing compound metabolic, etc. were the predominant terms belonging to the biological process. The significant enrichment terms in the molecular function were hydrogen ion transmembrane transporter activity, monovalent inorganic cation transmembrane transporter activity, cation transmembrane transporter activity, etc. For the down-regulated DEGs in the 3.5% ethanol group (Figure 2C), cellular protein metabolic process, organonitrogen compound metabolic process, and organonitrogen biosynthetic process were the most abundant in the biological process. Structural constituent of ribosome, structural molecule activity, and RNA binding were the most abundant in the molecular function. For the up-regulated DEGs in this group (Figure 2D), carbohydrate metabolic process, single-organism catabolic process, and single-organism carbohydrate metabolic process were the main terms belonging to the biological process. Hydrolase activity, cofactor binding, FMN binding, etc. were the main enrichment terms in molecular function.

**FIGURE 3:**
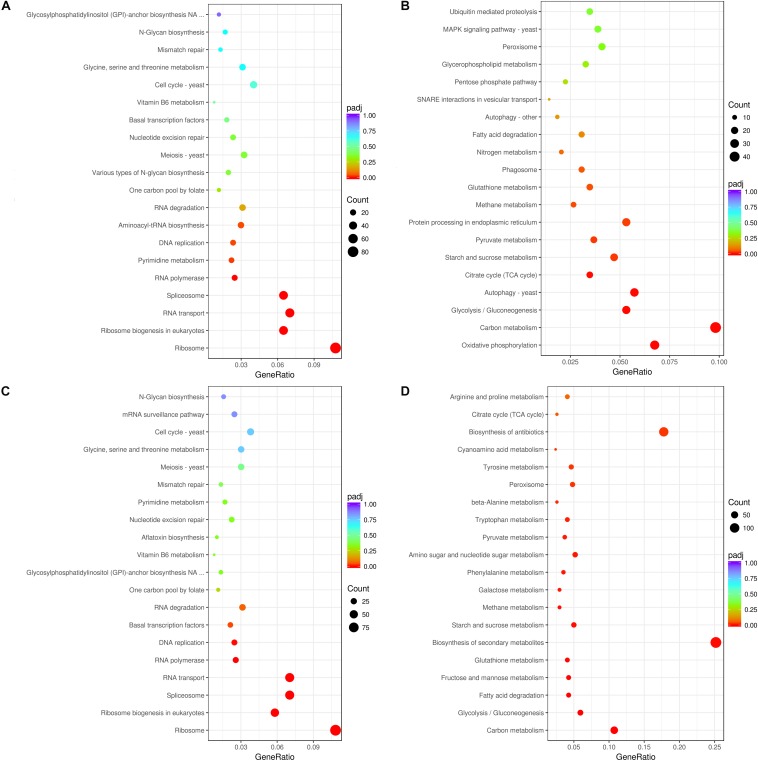
KEGG enrichment of down-regulated **(A,C)** and up-regulated **(B,D)** DEGs. **(A,B)** The ordinate means with 2.5% ethanol treatment. **(C,D)** The ordinate means with 3.5% ethanol treatment. The size of the plot represents the number of DEGs in one GO term; the color of the plot close to red represents more significant enrichment.

In *A. flavus* treated with 2.5% ethanol, the top 20 enriched KEGG pathway were shown in Figures 3A,B. For the down-regulated DEGs, the most abundant genes (83 DEGs) were enriched in ribosome (afv03010), and 54, 50, and 50 DEGs were enriched in RNA transport (afv03013), ribosome biogenesis (afv03008), and spliceosome (afv03040), respectively. For the up-regulated DEGs (Figure 3B), the most abundant genes (48 DEGs) were enriched in carbon metabolism (afv01200), and 33, 28, 26, and 26 DEGs were enriched in oxidative phosphorylation (afv00190), autophagy-yeast (afv04138), glycolysis/gluconeogenesis (afv00010), and protein processing in endoplasmic reticulum (afv04141), respectively. For the 3.5% ethanol group, the most abundant down-regulated DEGs (Figure 3C) were enriched in ribosome (afv03010, 97 DEGs), and 63, 63, and 52 DEGs were enriched in spliceosome (afv03008), RNA transport (afv03013), and ribosome biogenesis in eukaryotes (afv03008), respectively. The most abundant up-regulated DEGs (Figure 3D) were enriched in biosynthesis of secondary metabolites (afv01110, 136 DEGs), and 96, 58, and 32 DEGs were enriched in biosynthesis of antibiotics (afv01130), carbon metabolism (afv01200), and glycolysis/gluconeogenesis (afv00010), respectively.

### Expression Analysis of Pigment (#10), Aflatrem (#15), Aflatoxin (#54), and Cyclopiazonic Acid (#55) Biosynthesis Genes in Response to Ethanol

As shown in Table 1, in pathway #10, AFLA_016120 encoding an O-methyltransferase family protein and AFLA_016130 were down-regulated by 2.5% ethanol, but all three genes in this pathway were up-regulated by 3.5% ethanol. In pathway #15, the expression levels of most genes were very low except for AFLA_045450. In pathway #55, AFLA_139470 encoding a FAD-dependent oxidoreductase, AFLA_139480 encoding a tryptophan dimethylallyl transferase, and AFLA_139480 encoding a hybrid PKS/NRPS enzyme were down-regulated by 2.5% ethanol, while AFLA_139460 coding a MFS multidrug transporter was up-regulated. Under 3.5% ethanol treatment, four genes in pathway #55 were all down-regulated. In AF pathway #54, *aflLa* (a similar hypothetical gene of *aflL*), and *aflG* were up-regulated by 2.5% ethanol, while *aflYd* and *aflYb* (*aflYa-e* are genes in sugar cluster and the last letters indicate the sequence of genes in the cluster) were down-regulated. The expression changes of other genes in pathway #54 were slight after 2.5% ethanol treatment. Interestingly, all of AF cluster genes were down-regulated by 3.5% ethanol except for *aflC*. The two key regulator genes *aflR* and *aflS* were both down-regulated by 3.5% ethanol compared to the control with log_2_FC values −1.31 and −1.73, respectively. For the structural genes, the expression of *aflK* and *aflLa* was completely inhibited, and *aflV*, *aflP*, *aflO*, *aflL*, and *aflM* were markedly down-regulated with log_2_FC values ≤ −10, and *aflY*, a*flX*, *aflW*, *aflQ*, *aflI*, *aflG*, *aflN*, *aflMa*, *aflE*, and *aflJ* were down-regulated with log_2_FC values ≤ −5. It is worth mentioning that *aflY(a–d)* genes belong to the sugar cluster and most of them appear to be more down-regulated when 2.5% ethanol was applied. However, the *aflYa* gene encoding NADH oxidase was significantly down-regulated by 3.5% ethanol, while the other four genes did not change significantly.

**TABLE 1:** Transcriptional activity of genes in the biosynthesis of conidial pigment (#10), aflatrem (#15), aflatoxin (#54), and cyclopiazonic acid (#55).

**Cluster ID**	**Gene ID (AFLA_x)**	**CK^∗^ (FPKM)**	**E2.5^∗^ (FPKM)**	**E3.5^∗^ (FPKM)**	**E2.5^∗^ Log**	**E3.5^∗^ Log**	**Annotated_gene_function**
10	AFLA_016120	6.91	3.54	15.79	–0.95	1.15	O-methyltransferase family protein
10	AFLA_016130	4.02	1.64	8.55	–1.29	1.05	Hypothetical protein
10	AFLA_016140	25.18	29.44	98.18	0.23	1.92	Conidial pigment biosynthesis scytalone dehydratase Arp1
15	AFLA_045450	26.95	38.23	45.50	0.51	0.72	Ankyrin repeat-containing protein, putative
15	AFLA_045460	0.63	1.56	3.25	1.32	2.28	Hypothetical protein
15	AFLA_045470	0.05	0.03	0.12	–0.65	1.23	Non-sense-mediated mRNA decay protein, putative
15	AFLA_045480	0.00	0.00	0.09	/	/	Conserved hypothetical protein
15	AFLA_045490	0.09	0.02	0.26	–1.91	1.55	Dimethylallyl tryptophan synthase, putative
15	AFLA_045500	0.24	0.18	0.89	–0.35	1.88	Cytochrome P450, putative
15	AFLA_045510	0.13	0.19	0.23	0.54	0.77	integral membrane protein
15	AFLA_045520	0.06	0.00	0.09	/	/	Integral membrane protein
15	AFLA_045530	0.10	0.21	0.33	1.08	1.73	Conserved hypothetical protein
15	AFLA_045540	0.03	0.21	0.20	2.89	2.77	Cytochrome P450, putative
15	AFLA_045550	0.86	0.10	0.41	–3.18	–1.08	Hypothetical protein
15	AFLA_045560	2.67	0.48	0.85	–2.48	–1.69	Carboxylic acid transport protein
15	AFLA_045570	0.62	15.47	1.71	4.64	1.41	Acetyl xylan esterase, putative
54	AFLA_139100	1.14	0.61	1.84	–0.89	0.66	*aflYe/orf*/Ser-Thr protein phosphatase family protein
54	AFLA_139110	0.86	0.38	0.77	–1.14	–0.19	*aflYd/sugR*/sugar regulator
54	AFLA_139120	1.02	0.62	1.17	–0.72	0.16	*aflYc/glcA*/glucosidase
54	AFLA_139130	5.30	2.15	3.57	–1.30	–0.61	*aflYb/hxtA*/putative hexose transporter
54	AFLA_139140	14.65	16.49	0.33	0.18	–5.48	*aflYa/nadA*/NADH oxidase
54	AFLA_139360	80.84	81.82	33.67	0.02	–1.31	a*flR/apa-2/afl-2/*transcription activator
54	AFLA_139340	116.22	66.04	35.10	–0.81	–1.78	*aflS*/pathway regulator
54	AFLA_139150	60.40	61.41	0.74	0.03	–6.39	*aflY/hypA/hypP*/hypothetical protein
54	AFLA_139160	104.46	63.29	2.85	–0.72	–5.23	*aflX/ordB*/monooxygenase/oxidase
54	AFLA_139170	56.80	49.75	0.50	–0.19	–6.86	*aflW/moxY*/monooxygenase
54	AFLA_139180	54.39	69.01	0.04	0.35	–10.28	*aflV/cypX*/cytochrome P450 monooxygenase
54	AFLA_139190	38.21	51.44	0	0.43	Down	*aflK/vbs*/VERB synthase
54	AFLA_139200	6.99	8.62	0.01	0.31	–9.58	*aflQ/ordA/ord-1*/oxidoreductase/cytochrome P450 monooxigenase
54	AFLA_139210	25.03	38.34	0.01	0.62	–10.92	*aflP/omtA/omt-1*/O-methyltransferase A
54	AFLA_139220	52.83	43.14	0.03	–0.29	–10.83	*aflO/omtB/dmtA*/O-methyltransferase B
54	AFLA_139230	5.24	9.54	0.03	0.87	–7.17	*aflI/avfA*/cytochrome P450 monooxygenase
54	AFLA_139240	20.69	47.89	0	1.22	Down	*aflLa/hypB*/hypothetical protein
54	AFLA_139250	46.25	52.77	0.03	0.20	–10.53	*aflL/verB*/desaturase/P450 monooxygenase
54	AFLA_139260	13.18	32.24	0.07	1.29	–7.40	*aflG/avnA/ord-1*/cytochrome P450 monooxygenase
54	AFLA_139270	744.25	461.76	51.46	–0.68	–3.90	*aflNa/hypD*/hypothetical protein
54	AFLA_139280	23.45	19.62	0.24	–0.25	–6.57	*aflN/verA*/monooxygenase
54	AFLA_139290	140.71	177.05	0.35	0.34	–8.66	*aflMa/hypE*/hypothetical protein
54	AFLA_139300	479.94	507.79	0.09	0.09	–12.46	*aflM/ver-1*/dehydrogenase/ketoreductase
54	AFLA_139310	104.67	119.71	0.78	0.20	–7.10	*aflE/norA/aad/adh-2*/NOR reductase/dehydrogenase
54	AFLA_139320	169.61	176.89	4.63	0.07	–5.24	*aflJ/estA*/esterase
54	AFLA_139330	263.26	286.95	9.08	0.13	–4.90	*aflH/adhA*/short chain alcohol dehydrogenase
54	AFLA_139370	25.57	24.00	10.68	–0.09	–1.31	*aflB/fas-1*/fatty acid synthase beta subunit
54	AFLA_139380	7.60	9.94	3.98	0.39	–0.98	*aflA/fas-2/hexA*/fatty acid synthase alpha subunit
54	AFLA_139390	101.40	127.23	4.63	0.33	–4.50	*aflD/nor-1/*reductase
54	AFLA_139400	41.13	73.10	3.82	0.83	–3.47	*aflCa/hypC*/hypothetical protein
54	AFLA_139410	5.11	8.34	5.59	0.71	0.09	*aflC/pksA/pksL1/*polyketide synthase
54	AFLA_139420	82.13	98.69	41.71	0.27	–1.02	*aflT/aflT*/transmembrane protein
54	AFLA_139430	9.30	8.65	8.15	–0.10	–0.23	*aflU/cypA/*P450 monooxygenase
54	AFLA_139440	37.20	29.33	18.42	–0.34	–1.06	*aflF/norB/*dehydrogenase
55	AFLA_139460	659.14	1823.80	260.75	1.47	–1.38	MFS multidrug transporter, putative
55	AFLA_139470	30.56	18.67	7.29	–0.71	–2.11	FAD dependent oxidoreductase, putative
55	AFLA_139480	45.38	23.43	16.66	–0.95	–1.49	Dimethylallyl tryptophan synthase, putative
55	AFLA_139490	0.49	0.11	0.28	–2.17	–0.84	Hybrid PKS/NRPS enzyme, putative

*^∗^CK, Control; E2.5, 2.5% ethanol; E3.5, 3.5% ethanol.*

**FIGURE 4:**
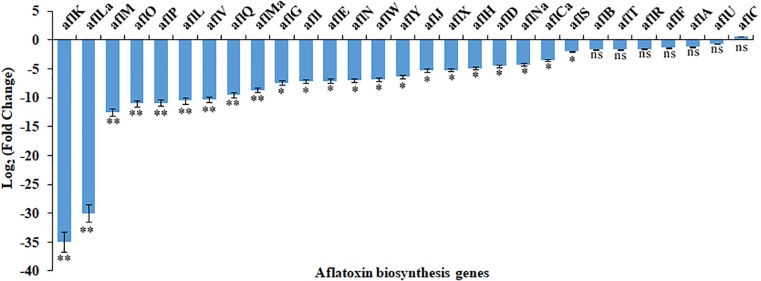
The differential expression of genes in aflatoxin biosynthesis cluster in response to 3.5% ethanol. ns, not significant; ^∗^*p* < 0.05; ^∗∗^*p* < 0.01.

The RNA-seq results were confirmed by analyzing the expression of AF cluster genes in *A. flavus* treated with 3.5% ethanol using qRT-PCR method. As shown in Figure 4, the expression mode of these genes was consistent with the RNA-seq data.

### Genes Involved in the Development

The transcription levels of genes involved in development are shown in Supplementary Table S1. From the expression profile data, we found that some genes involved in conidiophores development including *FlbA*, *FlbC*, *FlbD*, and *HymA* were down-regulated by 2.5 and 3.5% ethanol. For the velvet complex, *VeA* was up-regulated by 2.5% ethanol, but was down-regulated by 3.5% ethanol. *FluG* (AFLA_039530) and *VosA* were down-regulated by 2.5 and 3.5% ethanol. However, *LaeA* did not show a significant differential expression with ethanol treatment. *AbaA* controlling phialide differentiation, development regulator *Mod-A* (AFLA_009340), and conidial hydrophobin *RodB* were down-regulated by 2.5 and 3.5% ethanol. The *BrlA* mediating conidiophores was up-regulated by 3.5% ethanol.

### Genes Involved in Fungal Oxidative Stress

The expression levels of genes involved in oxidative stress response are shown in Supplementary Table S2. The catalase/peroxidase/superoxide dismutase genes were all significantly modulated by ethanol. The expression of *Cat1*, *Cat2*, *CatA*, and *sod1* were up-regulated by 2.5 and 3.5% ethanol while mnSOD was down-regulated. The transcriptional levels of *Cat* were down-regulated by 2.5% ethanol, but were up-regulated by 3.5% ethanol. The bZIP transcription factor *ap-1* and two C_2_H_2_ transcription factors *msnA* and *mtfA* were up-regulated by 2.5 and 3.5% ethanol. However, the stress response transcription factor *srrA* was down-regulated by 2.5 and 3.5% ethanol. The MAP kinase *sakA* gene was obviously down-regulated by 2.5 and 3.5% ethanol. The transcriptional level of fatty acid oxygenase *ppoA* was down-regulated by 2.5 and 3.5% ethanol, but *ppoC* was up-regulated. Meantime, *ppoB* was expressed at a very low level. The expression of GPCRs *gprC*, *gprH*, *gprM*, *gprR*, and *gprS* was down-regulated by 2.5 and 3.5% ethanol, while that of *gprD* and *gprG* was up-regulated. The transcriptional level of *gprK* was down-regulated by 2.5% ethanol, but was up-regulated by 3.5% ethanol.

### Genes Involved in Metabolism of Ethanol

**FIGURE 5:**
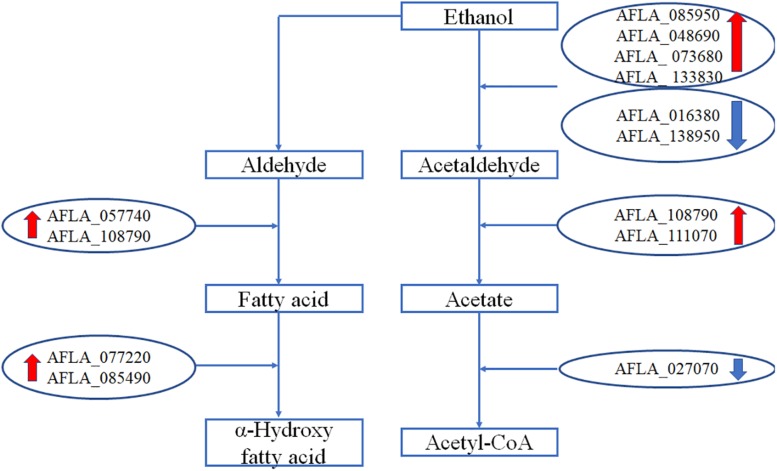
The differential expression of genes involved in metabolism of ethanol.

The expression levels of genes involved in metabolism of ethanol are shown in Figure 5. After treatment with 3.5% ethanol, most of the genes involved in the metabolism of ethanol were up-regulated except for the two alcohol dehydrogenase genes, AFLA_016380 and AFLA_138950, involved in the process converting ethanol to acetaldehyde and the acetate and CoA ligase gene AFLA_027070 involved in the conversion of acetate to acetyl-CoA. The four alcohol dehydrogenase genes AFLA_085950, AFLA_048690, AFLA_073680, and AFLA_0133830 were up-regulated by 3.5% ethanol with Log_2_FC values of 2.94, 1.48, 2.82, and 1.54, respectively. The two aldehyde dehydrogenase *AldA* genes were up-regulated by 3.5% ethanol with Log_2_FC values of 2.33 and 1.69, respectively. The NADPH flavin oxidoreductase gene AFLA_077220 and P450 family fatty acid hydroxylase AFLA_085490 involved in the conversion of fatty acid to α-hydroxy fatty acid were up-regulated by 3.5% ethanol with Log_2_FC of 1.65 and 1.86, respectively.

## Discussion

AF biosynthesis needs more than 23 enzymatic reactions (Cleveland et al., 2009). In *A. flavus*, the genes encoding these enzymes are located in an AF pathway gene cluster and are regulated by AFLR and AFLS (Bhatnagar et al., 2003; Cleveland et al., 2009). In our RNA-Seq data, the transcriptional level changes of the AF cluster genes were stronger in *A. flavus* treated with 3.5% ethanol compared to 2.5% ethanol. Of 30 AF cluster genes, the expression of 27 genes was significantly down-regulated by 3.5% ethanol except for *aflA*, *aflC*, and *aflU*. It is important to notice that the two key regulator genes *aflR* and *aflS* were both down-regulated by 3.5% ethanol, together with the down-regulation of the structural genes in the cluster. The gene *aflK*, encoding a versicolorin (VERB) synthase involved in conversion of versiconal (VAL) to VERB (McGuire et al., 1996; Silva and Townsend, 1996; Silva et al., 1996), was completely inhibited. This conversion is a critical step in AF biosynthesis because it closes the bifuran ring of AFs, which is a prerequisite for binding to DNA and gives AFs the mode of action as a mutagen (Yu et al., 2004). In addition, the expression of *aflLa/hypB*, a hypothetical protein gene, was also completely inhibited by 3.5% ethanol. Similarly, Lin et al. (2013) found that *aflLa/hypB* was completely inhibited by 5-azacytidine (5-AC), an inactivator of DNA methyltransferase. It was reported that *aflLa/hypB* might be involved in the second oxidation step converting O-methylsterigmatocystin (OMST) to a 7-membered ring lactone, the precursor for AFB_1_ formation (Ehrlich, 2009). Our previous study indicated that *aflLa/hypB* was one of the target genes for rapid identification of atoxigenic strains (Wei et al., 2014). These findings suggested that 3.5% ethanol inhibited AF biosynthesis by down-regulating the transcriptional levels of transcriptional factor *aflR*, the cofactor *aflS*, and subsequently most of the structural genes.

As a general cell toxic substance, ethanol affects the integrity of the cell membrane, inactivates cellular enzymes, and destroys protein structure, leading to the inhibition of fungal growth, viability, and conidia formation (Ma et al., 2019). In addition, ethanol triggered internal cellular perturbations like irregular nuclei and the aggregation of scattered vacuoles in fungal cells. The abovementioned disorders of cellular functions in turn could lead to the reduction of AFs biosynthesis. Moreover, ethanol also influenced the transcription levels of some global regulator factors. The velvet family proteins VeA, VelB, and LaeA of *A. flavus* form a heterotrimeric velvet complex to coordinate sexual development and biosynthesis of several secondary metabolites in the dark (Bayram et al., 2008; Chang et al., 2013). The coordinating and balanced interactions among the velvet family proteins together with FluG play a key role in maintaining programmed AFs biosynthesis and conidiation and sclerotial production (Chang et al., 2013). After treatment with 3.5% ethanol, the expression of *veA* and *fluG* was significantly down-regulated with Log2FC −2.97 and −4.03, respectively. The down-regulation of *veA* suppressed the expression of several key AFs genes including *aflR*, *aflD*, *aflM*, and *aflP* and resulted in the inhibition of AF biosynthesis (Duran et al., 2007).

The oxidative stress was recognized as a prerequisite for AFs formation in *A. flavus* and *A. parasiticus* (Reverberi et al., 2008; Zhang et al., 2015; Lv et al., 2018; Guan et al., 2019). In the meantime, AFs biosynthesis is thought to protect the fungus against oxidative stress (Wang et al., 2019). Several previous studies have indicated that some AFs inhibitors can regulate the stress response system of fungi (Reverberi et al., 2005; Grintzalis et al., 2014; Sun et al., 2015; Caceres et al., 2017). After treatment with 3.5% ethanol, all catalase genes including *Cat*, *Cat1*, *Cat2*, *CatA*, and Cu, Zn superoxide dismutase gene *sod1* were up-regulated, while only Mn superoxide dismutase gene *mnSOD* was down-regulated. Similarly, piperine exposure significantly induced decreased expression of *veA* together with the overexpression of several bZIP transcription factors genes like *atfA*, *atfB*, and *ap-1* and genes encoding catalase such as *catA*, *cat2*, and superoxide dismutase like *sod1* in *A. flavus* (Caceres et al., 2017). Moreover, this gene response was coupled with an obvious increase of catalase enzymatic activity (Caceres et al., 2017). Cinnamaldehyde exposure resulted in the up-regulation of several transcription factors genes like *srrA*, *msnA*, and *atfB* and genes encoding catalase like *cat*, *cat1*, *catA*, and superoxide dismutase including *sod1* and *mnSOD* (Wang et al., 2019).

The transcriptional levels of genes involved in the antioxidant system were modulated by the upstream transcription factors including *ap-1*, *atfA*, *atfB*, *msnA*, *mtfA*, and *PacC* (Hong et al., 2013). As a redox-state sensor protein, the functions of Ap-1 are highly conserved in yeast, fungi, and mammals (Toone et al., 2001; Caceres et al., 2017). In fungi, the *N*- and *C*-terminal cysteine-rich domains of Ap-1-like protein might act as a sensor target of reactive oxygen species (ROS) like H_2_O_2_ (Sies, 2014). In *A. parasiticus*, the deletion of *ApyapA* causes the increase of AFs biosynthesis, oxidative stress, premature conidiogenesis, and an earlier transcription of AFs cluster genes like *aflR* and *aflE* (Reverberi et al., 2008; Caceres et al., 2017). The bZIP transcription factor SrrA, an ortholog of *Saccharomyces cerevisiae* Skn7 and *Saccharomyces pombe* Prr1, mediates cellular response to environmental stimuli (Hagiwara et al., 2007; Vargas-Perez et al., 2007). In *A. parasiticus*, Hong et al. (2013) identified a recognition site of SrrA in promoters of the antioxidant genes *cat1* and *mnsod*, and AFs biosynthetic genes *aflB* (*fas-1*) and *aflM* (*ver-1*). Moreover, the adjacent binding sites of SrrA and AP-1 in the promoter suggest that they can interact and are involved in the transcriptional regulation of AFs genes (Hong et al., 2013). In the present study, an up-regulation of *ap-1* and a down-regulation of *srrA* were observed upon 3.5% ethanol addition. MsnA is a C_2_H_2_ zinc finger transcription factor and can respond to some cellular stress such as oxidative stress, carbon starvation, heat shock, and osmotic stress (Martinez-Pastor et al., 1996; Hong et al., 2013). In *A. flavus* and *A. parasiticus*, disruption of *msnA* led to increased AFs biosynthesis and the production of conidia, ROS, and kojic acid, although fungal growth was inhibited (Chang et al., 2011). In addition, *msnA* deletion down-regulated transcription levels of genes encoding antioxidant enzymes, which protect fungus against ROS (Hong et al., 2013). Our previous studies revealed that eugenol and cinnamaldehyde up-regulated the expression of *msnA* and inhibited AFs biosynthesis (Lv et al., 2018; Wang et al., 2019). A similar finding, the up-regulation of *msnA* in *A. flavus* treated with 3.5% ethanol, was obtained in the present study. MtfA is another C_2_H_2_ zinc finger transcription factor, which was originally identified in *Aspergillus nidulans* and was involved in sterigmatocystin (ST) regulation (Ramamoorthy et al., 2013). The disruption and overexpression of *mtfA* both induced the decreased production of ST (Zhuang et al., 2016). In *A. flavus*, overexpression of *mtfA* dramatically reduced AFB_1_ production accompanied by a drastic reduction of *aflR* expression compared to the WT strain while deletion of *mtfA* did not significantly influenced AFB_1_ production (Zhuang et al., 2016). Caceres et al. (2016) indicated that eugenol up-regulated the expression of *mtfA* and inhibited AFB_1_ production. Similarly, the transcription level of *mtfA* was up-regulated by 3.5% ethanol in the present study.

It is important to point out that the transcriptional status is very fluctuating depending on transcription rate and half-life of the mRNA, which may be very short compared to the more accumulative and stable concentration of the AF produced. This means that the transcription may not be directly correlated with the amount of AF produced at each time point. Therefore, the following mechanism of action of ethanol on the inhibition of AFs proposed in this study is based on the RNA-seq data on the 7th day.

**FIGURE 6:**
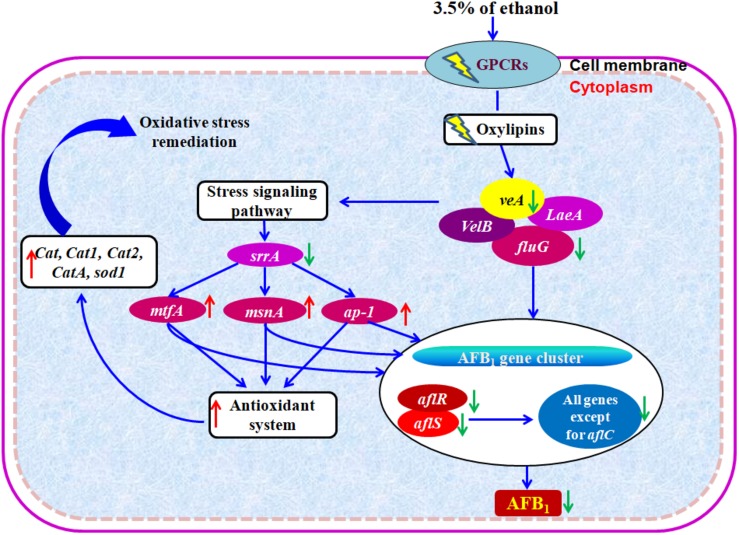
Hypothetical mechanism of action of ethanol on AFB_1_ biosynthesis. Down- and up-regulation of gene on ethanol addition is expressed using green and red arrows, respectively.

Based on the abovementioned results, we proposed a hypothetical mechanism of action of ethanol on the inhibition of AFs (Figure 6). Taken together, the enhanced transcription levels of the stress response system, such as bZIP transcription factor *ap-1*, C_2_H_2_ transcription factors *msnA* and *mtfA*, the down-regulation of stress response transcription factor *srrA*, and the overexpression of genes encoding for antioxidant system including catalase genes and superoxide dismutase gene in *A. flavus* treated with ethanol, significantly down-regulate the expression of AF biosynthesis genes and in turn result in the inhibition of AFs production.

## Conclusion

In the present study, we reveal the transcription modulation mechanism behind ethanol’s AFB_1_-repressing action using an RNA-Seq. The RNA data indicated that (1) with ethanol treatment, AFB_1_ cluster genes were dramatically down-regulated following the up-regulation of their specific regulators *aflS/aflR*; (2) ethanol’s mechanism of action involved the down-regulation of the global regulator *veA* and *fluG*; (3) ethanol’s transcription modulation mechanism involved the decreased expression of stress response transcription factor *srrA* together with overexpression of bZIP transcription factor *ap-1* and C_2_H_2_ transcription factors *msnA* and *mtfA*; (4) ethanol induced enhanced levels of anti-oxidant enzymatic genes including *Cat*, *Cat1*, *Cat2*, *CatA*, and Cu, Zn superoxide dismutase gene *sod1*. In conclusion, these results strongly suggest that ethanol inhibits AFB_1_ biosynthesis by *A. flavus* via enhancing fungal oxidative stress response.

## Data Availability Statement

The datasets generated for this study can be found in the https://www.ncbi.nlm.nih.gov/Traces/study/?acc=PRJNA558521.

## Author Contributions

FX and QY conceived and designed the experiments. YR, JJ, and MZ performed the experiments. YR and FX analyzed the data and wrote the manuscript.

## Conflict of Interest

The authors declare that the research was conducted in the absence of any commercial or financial relationships that could be construed as a potential conflict of interest.
