# Supplementary material for: Ethanol Inhibits Aflatoxin B1 Biosynthesis in Aspergillus flavus by Up-Regulating Oxidative Stress-Related Genes
Source: Front Microbiol. 2020 Jan 17;10:2946. doi: 10.3389/fmicb.2019.02946 (PMC6978751; doi:10.3389/fmicb.2019.02946)
Supplement: Supplementary file 1 [file Data_Sheet_1.docx]

Title:

**Ethanol Inhibits Aflatoxin B_1_ Biosynthesis in *Aspergillus flavus* by Up-regulating Oxidative Stress Related Genes**

Author names and affiliations:

Yaoyao Ren^1^, Jing Jin^2^, Mumin Zheng^1^, Qingli Yang^1^*, Fuguo Xing^1,2^*

1. College of Food Science and Engineering, Qingdao Agricultural University, Qingdao, 266109, P. R. China

2. Key Laboratory of Agro-products Quality and Safety Control in Storage and Transport Process, Ministry of Agriculture and Rural Affairs / Institute of Food Science and Technology, Chinese Academy of Agricultural Sciences, Beijing 100193, P. R. China

*Corresponding Author

**Qingli Yang**: College of Food Science and Engineering, Qingdao Agricultural University, 700 Changcheng Road, Qingdao, 266109, P. R. China

Tel: +86-0532-88030449

E-mail: rice407@163.com

**Fuguo Xing**: Institute of Food Science and Technology, Chinese Academy of Agricultural Sciences, 2 Yuanmingyuan West Road, Haidian District, Beijing 100193, P. R. China

Tel: +86-10-62811868

E-mail: [xingfuguo@caas.cn](mailto:xingfuguo@caas.cn)

TABLE S1 | Transcriptional activity of genes involved in *A. flavus* development.

| Gene_ID | CK (FPKM) | E2.5 (FPKM) | E3.5 (FPKM) | Log E2.5 | Log E3.5 | Annotated_gene_function |
| --- | --- | --- | --- | --- | --- | --- |
| AFLA_066460 | 149.60 | 1716.01 | 19.73 | 3.52 | -2.97 | developmental regulator *VeA* |
| AFLA_033290 | 19.00 | 27.19 | 12.05 | 0.52 | -0.70 | regulator of secondary metabolism *LaeA* |
| AFLA_081490 | 29.32 | 36.04 | 47.57 | 0.30 | 0.66 | nucleoside diphosphatase *Gda1/VelB* |
| AFLA_026900 | 17.77 | 6.75 | 10.06 | -1.39 | -0.87 | developmental regulator *VosA* |
| AFLA_039530 | 20.68 | 0.95 | 1.31 | -4.42 | -4.03 | *FluG* |
| AFLA_046990 | 173.50 | 132.78 | 125.18 | -0.38 | -0.51 | APSES transcription factor *StuA* |
| AFLA_136410 | 99.73 | 96.53 | 88.65 | -0.04 | -0.21 | transcriptional regulator *Medusa* |
| AFLA_020210 | 83.94 | 57.07 | 62.22 | -0.55 | -0.47 | sexual development transcription factor *NsdD* |
| AFLA_018340 | 106.42 | 87.71 | 50.29 | -0.27 | -1.12 | G-protein complex alpha subunit *GpaA*/*FadA* |
| AFLA_048650 | 43.97 | 29.79 | 28.89 | -0.56 | -0.65 | sexual development transcription factor *SteA* |
| AFLA_071090 | 61.82 | 244.20 | 59.36 | 1.99 | -0.09 | GTP-binding protein *EsdC* |
| AFLA_131490 | 67.73 | 36.20 | 28.21 | -0.90 | -1.31 | conserved hypothetical protein |
| AFLA_083110 | 126.17 | 117.70 | 98.25 | -0.10 | -0.41 | conidiation-specific protein (Con-10), putative |
| AFLA_024890 | 14.21 | 7.17 | 5.03 | -0.98 | -1.55 | cell differentiation and development protein *Fsr1*/*Pro11* |
| AFLA_134030 | 9.12 | 4.00 | 3.67 | -1.18 | -1.36 | developmental regulator *FlbA* |
| AFLA_137320 | 33.70 | 15.81 | 6.73 | -1.08 | -2.37 | C_2_H_2_ conidiation transcription factor *FlbC* |
| AFLA_080170 | 4.63 | 0.84 | 1.64 | -2.46 | -1.53 | MYB family conidiophore development protein *FlbD*, putative |
| AFLA_016140 | 25.18 | 29.44 | 98.18 | 0.23 | 1.92 | conidial pigment biosynthesis scytalone dehydratase *Arp1* |
| AFLA_079710 | 23.10 | 8.07 | 5.13 | -1.51 | -2.22 | conidiophore development protein *HymA* |
| AFLA_044800 | 38.94 | 34.51 | 16.65 | -0.17 | -1.27 | conidiation protein Con-6, putative |
| AFLA_101920 | 3.00 | 2.51 | 3.96 | -0.24 | 0.36 | extracellular developmental signal biosynthesis protein *FluG* |
| AFLA_052030 | 4.72 | 2.81 | 3.19 | -0.74 | -0.61 | developmental regulatory protein *WetA* |
| AFLA_029620 | 3.28 | 0.32 | 0.31 | -3.32 | -3.45 | transcription factor *AbaA* |
| AFLA_009340 | 3.87 | 0.21 | 0.34 | -4.22 | -3.54 | developmental regulator *Mod-A*, putative |
| AFLA_082850 | 0.23 | 0.33 | 0.62 | 0.55 | 1.40 | C_2_H_2_ type conidiation transcription factor *BrlA* |
| AFLA_014260 | 0.89 | 0.36 | 0.45 | -1.29 | -1.06 | conidial hydrophobin *RodB*/*HypB* |
| AFLA_006180 | 0.25 | 0.06 | 0.24 | -2.03 | -0.09 | conidial pigment biosynthesis oxidase *Arb2*/brown2 |
| AFLA_098380 | 0.20 | 0.62 | 0.37 | 1.58 | 0.77 | conidial hydrophobin *RodA*/*RolA* |
| AFLA_044790 | 668.23 | 1564.86 | 459.41 | 1.23 | -0.58 | conidiation-specific family protein |

* CK=Control; E2.5=2.5% ethanol; E3.5=3.5% ethanol.

TABLE S2 | Transcriptional involved in *A. flavus* of MAPK pathway, Oxylipins, and GPCRs genes

| Gene_ID | Gene | CK (FPKM) | E2.5 (FPKM) | E3.5 (FPKM) | Log  E2.5 | Log E3.5 | Annotated_gene_function |
| --- | --- | --- | --- | --- | --- | --- | --- |
| AFLA_062500 | *Maf1* | 69.75 | 63.84 | 49.12 | -0.12 | -0.55 | mitogen-activated protein kinase MAF1 |
| AFLA_083380 | *Pbs2* | 68.33 | 34.74 | 37.97 | -0.97 | -0.89 | MAP kinase kinase (Pbs2), putative |
| AFLA_048880 | *Ste11* | 8.65 | 7.62 | 5.71 | -0.18 | -0.64 | MAP kinase kinase kinase Ste11 |
| AFLA_103480 | *Ste7* | 6.10 | 5.97 | 6.92 | -0.03 | 0.13 | MAP kinase kinase Ste7 |
| AFLA_034170 | *Fus3* | 47.63 | 37.27 | 37.36 | -0.35 | -0.39 | MAP kinase FUS3/KSS1 |
| AFLA_035530 | *Ste20* | 17.54 | 10.42 | 15.59 | -0.74 | -0.21 | serine/threonine kinase Ste20 |
| AFLA_073630 | *Ste20-like* | 3.03 | 1.80 | 2.39 | -0.75 | -0.39 | serine/threonine protein kinase, Ste20-like |
| AFLA_021030 |  | 18.34 | 17.52 | 23.30 | -0.06 | 0.31 | serine/threonine protein kinase, putative |
| AFLA_052570 | *mpkA* | 41.22 | 48.33 | 43.54 | 0.24 | 0.04 | MAP kinase MpkA |
| AFLA_051240 | *Mkk2* | 134.80 | 104.93 | 163.50 | -0.36 | 0.24 | MAP kinase kinase (Mkk2), putative |
| AFLA_031560 | *bck1* | 25.85 | 27.22 | 21.79 | 0.08 | -0.29 | MAP kinase kinase kinase (Bck1), putative |
| AFLA_100250 | *Cat* | 0.16 | 0.04 | 0.64 | -2.01 | 1.91 | catalase Cat |
| AFLA_090690 | *Cat1* | 80.22 | 303.33 | 605.04 | 1.92 | 2.88 | mycelial catalase Cat1 |
| AFLA_122110 | *Cat2* | 3.43 | 55.34 | 19.46 | 4.02 | 2.46 | bifunctional catalase-peroxidase Cat2 |
| AFLA_056170 | *CatA* | 62.30 | 113.22 | 242.49 | 0.86 | 1.92 | spore-specific catalase CatA |
| AFLA_099000 | *sod1* | 82.84 | 207.07 | 382.14 | 1.33 | 2.16 | Cu, Zn superoxide dismutase SOD1 |
| AFLA_033420 | *mnSOD* | 2145.70 | 1576.95 | 482.18 | -0.44 | -2.19 | Mn superoxide dismutase MnSOD |
| AFLA_031340 | *atfA* | 106.15 | 146.45 | 175.28 | 0.47 | 0.68 | bZIP transcription factor (AtfA), putative |
| AFLA_094010 | *atfB* | 97.96 | 118.21 | 82.29 | 0.28 | -0.29 | bZIP transcription factor (Atf21), putative |
| AFLA_129340 | *ap-1* | 67.41 | 151.40 | 158.52 | 1.17 | 1.20 | bZIP transcription factor AP-1 |
| AFLA_110650 | *msnA* | 44.91 | 111.09 | 89.11 | 1.31 | 0.95 | C_2_H_2_ transcription factor (Seb1) |
| AFLA_091490 | *mtfA* | 24.74 | 54.54 | 105.99 | 1.15 | 2.06 | C_2_H_2_ finger domain protein, putative |
| AFLA_030580 | *pacC* | 87.58 | 232.76 | 210.12 | 1.42 | 1.22 | C_2_H_2_ transcription factor PacC, putative |
| AFLA_034540 | *srrA* | 53.41 | 23.42 | 22.04 | -1.18 | -1.32 | stress response transcription factor SrrA/Skn7, putative |
| AFLA_062210 | *sskA* | 17.99 | 22.15 | 17.05 | 0.31 | -0.12 | response regulator, putative |
| AFLA_068590 | *sskB* | 13.84 | 7.58 | 9.36 | -0.86 | -0.61 | MAP kinase kinase kinase SskB, putative |
| AFLA_099500 | *sakA1* | 3.36 | 1.15 | 1.96 | -1.52 | -0.83 | MAP kinase SakA |
| AFLA_061090 | *sakA2* | 16.74 | 1.43 | 1.89 | -3.55 | -3.19 | MAP kinase SakA |
| AFLA_026790 | *ppoA* | 51.67 | 12.50 | 15.04 | -2.04 | -1.83 | fatty acid oxygenase PpoA, putative |
| AFLA_120760 | *ppoB* | 0.00 | 0.00 | 0.01 | NA | UP | fatty acid oxygenase, putative |
| AFLA_030430 | *ppoC* | 0.27 | 0.58 | 0.60 | 1.11 | 1.10 | fatty acid oxygenase PpoC, putative |
| AFLA_025100 | *gpdA* | 5592.40 | 28855.00 | 14346.48 | 2.37 | 1.32 | glyceraldehyde 3-phosphate dehydrogenase GpdA |
| AFLA_046760 | *gfdB* | 73.82 | 44.20 | 117.44 | -0.74 | 0.63 | glycerol 3-phosphate dehydrogenase (GfdB), putative |
| AFLA_060740 | *gprA* | 3.12 | 2.60 | 3.65 | -0.25 | 0.18 | mating-type alpha-pheromone receptor PreB |
| AFLA_061620 | *gprB* | 6.57 | 3.66 | 4.65 | -0.83 | -0.54 | a-pheromone receptor PreA |
| AFLA_074150 | *gprC* | 2.22 | 0.74 | 1.15 | -1.57 | -0.99 | conserved hypothetical protein |
| AFLA_135680 | *gprD* | 5.67 | 35.96 | 28.99 | 2.67 | 2.32 | G protein-coupled receptor GprD |
| AFLA_006880 | *gprF* | 36.84 | 46.63 | 25.74 | 0.35 | -0.56 | PQ loop repeat protein |
| AFLA_067770 | *gprG* | 12.00 | 88.57 | 92.81 | 2.89 | 2.92 | PQ loop repeat protein |
| AFLA_006920 | *gprH* | 1.48 | 0.49 | 0.42 | -1.61 | -1.88 | cAMP receptor-like protein, putative |
| AFLA_127870 | *gprJ* | 60.53 | 115.89 | 56.06 | 0.94 | -0.15 | vacuolar membrane PQ loop repeat protein |
| AFLA_009790 | *gprK* | 0.05 | 0.00 | 0.10 | -2.38 | 1.07 | conserved hypothetical protein |
| AFLA_075000 | *gprM* | 2.57 | 0.75 | 0.53 | -1.77 | -2.32 | conserved hypothetical protein |
| AFLA_032130 | *gprO* | 25.08 | 6.87 | 7.36 | -1.86 | -1.81 | hemolysin-III channel protein Izh2, putative |
| AFLA_088190 | *gprP* | 20.88 | 12.54 | 15.65 | -0.73 | -0.46 | IZH family channel protein (Izh3), putative |
| AFLA_023070 | *gprR* | 47.42 | 36.41 | 13.09 | -0.38 | -1.90 | integral membrane protein |
| AFLA_006320 | *gprS* | 34.41 | 9.25 | 4.93 | -1.89 | -2.85 | PQ loop repeat protein |
| AFLA_117970 | *nopA* | 2451.85 | 4400.75 | 3819.89 | 0.85 | 0.60 | opsin, putative |

* CK=Control; E2.5=2.5% ethanol; E3.5=3.5% ethanol.
